# Tetraspanin 8 is a novel regulator of ILK-driven β1 integrin adhesion and signaling in invasive melanoma cells

**DOI:** 10.18632/oncotarget.15084

**Published:** 2017-02-04

**Authors:** Manale El Kharbili, Clément Robert, Tiffany Witkowski, Emmanuelle Danty-Berger, Laetitia Barbollat-Boutrand, Ingrid Masse, Nicolas Gadot, Arnaud de la Fouchardière, Paul C McDonald, Shoukat Dedhar, François Le Naour, Françoise Degoul, Odile Berthier-Vergnes

**Affiliations:** ^1^ Université de Lyon, Lyon, France; ^2^ Université Lyon 1, Lyon, France; ^3^ CNRS, UMR5534, Centre de Génétique et de Physiologie Moléculaire et Cellulaire, Villeurbanne, France; ^4^ Department of Dermatology, University of Colorado, Aurora, Colorado, USA; ^5^ Clermont Université, Université d’Auvergne, Imagerie Moléculaire et Thérapie Vectorisée, Clermont-Ferrand, France; ^6^ Inserm, U990, Clermont-Ferrand, France; ^7^ Laboratoire CarMeN (INSERM 1060, INRA1397, INSA), Université Lyon 1, Lyon, France; ^8^ Université Lyon 1, Fédération de Recherche Santé Lyon-Est, ANIPATH, Faculté Laennec, Lyon, France; ^9^ Département de Biopathologie, Centre Léon Bérard, Lyon, France; ^10^ Department of Integrative Oncology, British Columbia Cancer Research Center, Vancouver, Canada; ^11^ INSERM U602, Villejuif, France; ^12^ INSERM U1193, Hôpital Paul Brousse, Villejuif, France

**Keywords:** melanoma, matrix, integrin, tetraspanin 8, ILK

## Abstract

Melanoma is well known for its propensity for lethal metastasis and resistance to most current therapies. Tumor progression and drug resistance depend to a large extent on the interplay between tumor cells and the surrounding matrix. We previously identified Tetraspanin 8 (Tspan8) as a critical mediator of melanoma invasion, whose expression is absent in healthy skin. The present study investigated whether Tspan8 may influence cell-matrix anchorage and regulate downstream molecular pathways leading to an aggressive behavior. Using silencing and ectopic expression strategies, we showed that Tspan8-mediated invasion of melanoma cells resulted from defects in cell-matrix anchorage by interacting with β1 integrins and by interfering with their clustering, without affecting their surface or global expression levels. These effects were associated with impaired phosphorylation of integrin-linked kinase (ILK) and its downstream target Akt-S473, but not FAK. Specific blockade of Akt or ILK activity strongly affected cell-matrix adhesion. Moreover, expression of a dominant-negative form of ILK reduced β_1_ integrin clustering and cell-matrix adhesion. Finally, we observed a tumor-promoting effect of Tspan8 *in vivo* and a mutually exclusive expression pattern between Tspan8 and phosphorylated ILK in melanoma xenografts and human melanocytic lesions. Altogether, the *in vitro, in vivo* and *in situ* data highlight a novel regulatory role for Tspan8 in melanoma progression by modulating cell-matrix interactions through β1 integrin-ILK axis and establish Tspan8 as a negative regulator of ILK activity. These findings emphasize the importance of targeting Tspan8 as a means of switching from low- to firm-adhesive states, mandatory to prevent tumor dissemination.

## INTRODUCTION

Melanoma is the leading cause of all skin cancer related deaths, most likely due to its aggressiveness and resistance to current therapies. Despite all prevention efforts, its incidence is increasing faster than any other cancers [1]. During melanoma progression, tumor cells first grow within the epidermis, then acquire the ability to cross the epidermal basement membrane, invade the dermis and circulate to disseminate into distant organs [1]. All these steps require making and breaking contacts between melanoma cells and matrix components and depend to a large extent on transmembrane receptors belonging to the integrin family [2]. Integrins are composed of non-covalently linked α and β subunits that transduce bi-directional signals into and out of the cell [2]. Changes in integrin expression and/or function are associated with tumor progression through alteration of their crosstalk with the surrounding microenvironment [3, 4]. In particular, β1 integrins contribute to the aggressive behavior of cancer cells, resistance to chemo/radiotherapy [4] and targeted therapies, including melanoma [5].

Extracellular ligand binding leads to integrin clustering at cell-matrix contacts and subsequent recruitment of a complex heterogeneous class of proteins, including focal adhesion (FAK) and integrin linked (ILK) kinases [6], which signal through the MEK/ERK and PI3K/Akt pathways, both critical in melanoma progression [7]. Targeting upstream effectors of ERK with pharmacologic inhibitors yielded promising results [8] and clinical trials with combined PI3K and BRAF inhibitors, reported to inhibit melanoma tumor growth in mice [9], are currently in progress [10]. However, these targeted therapies lead to short-term clinical benefit and nearly all patients relapse due to acquired resistance [9, 10]. Recently, it has been shown that integrin β1-mediated matrix adhesion and signaling can drive melanoma resistance to BRAF inhibition [5]. Therefore, a deep understanding on how the integrin functions are modulated is mandatory to propose more efficient therapeutic strategies against melanoma.

Some members of the tetraspanin family have emerged as important regulators of expression levels, trafficking or post-translational modification of laminin-binding integrins [11, 12]. Tetraspanins associate laterally with one another and cluster dynamically with a large variety of transmembrane and signal-transducing partners, forming specialized membrane microdomains called “tetraspanin web” [13, 14]. Disorganization of these networks causes various diseases, including cancer, where tetraspanins can act as suppressors or promoters of metastasis [15, 16]. Unlike other tetraspanins, Tspan8 is expressed in a limited number of normal tissues [16] and its mechanism of action in modulating tumor progression is still poorly documented. Tspan8 overexpression in human carcinomas has been suggested to play a role in tumor progression [15, 16] since forced Tspan8 expression in various cell lines facilitated metastasis in mice [17–19]. The pro-migratory function of Tspan8 in epithelial cancer relies on its interaction with several adhesion molecules such as E-cadherin, EpCAM, claudin-7 and CD44 [15, 16]. Ectopic expression Tspan8 in Isreco colon cancer cell line stimulated cell motility through cooperation with the E-cadherin/p120-catenin membrane complex [20]. Conversely, Tspan8 silencing attenuated the migration of colon cancer cells and upregulated calcium-dependent cell-cell aggregation [21]. Overall, Tspan8 mediates the loss of intercellular connections between malignant epithelial cells, crucial for metastasis [22], whereas its contribution to cell-matrix anchorage remains obscure and has never been investigated in non-epithelial cancers. We previously reported that Tspan8 is a hallmark of melanoma progression, responsible for the acquisition of an invasive phenotype [23]. Unlike carcinomas where Tspan8 is expressed in normal cells and upregulated in tumor cells, Tspan8 is absent at the mRNA and protein levels in normal melanocytes and non-invasive melanoma cell lines, whereas it is strongly expressed in melanoma cells from a panel of invasive cell lines [23]. Accordingly, Tspan8 is undetectable in healthy skin and exclusively expressed in primary melanomas and lymph node metastases [23]. Since aggressive melanoma cells acquire the ability to invade surrounding tissues by changing their interactions with the local environment, the present study investigated whether Tspan8 may influence cell-matrix interactions and regulate downstream targets, leading to an aggressive behaviour.

We showed that the function of Tspan8 was unique as compared to any other. Indeed, Tspan8 impairs integrin-mediated anchorage of melanoma cells to matrix components by negatively regulating ILK activity, leading to the inhibition of its downstream target Akt-S473 and β1 integrin clustering. Moreover, Tspan8 promotes *in vivo* orthotopic tumor growth and an inverse pattern of Tspan8 and P-ILK expression was observed in melanoma xenografts as well as in human melanocytic lesions, underscoring the ability of Tspan8 to modulate ILK function in cutaneous microenvironment. Thus, our study identified Tspan8 as a novel regulator of ILK-driven β1 integrin signaling, mediating low interactions of melanoma cells with the surrounding extracellular microenvironment, mandatory for tumor escape.

## RESULTS

### Tspan8 expression markedly reduces melanoma cell anchorage to matrix components

**Figure 1:**
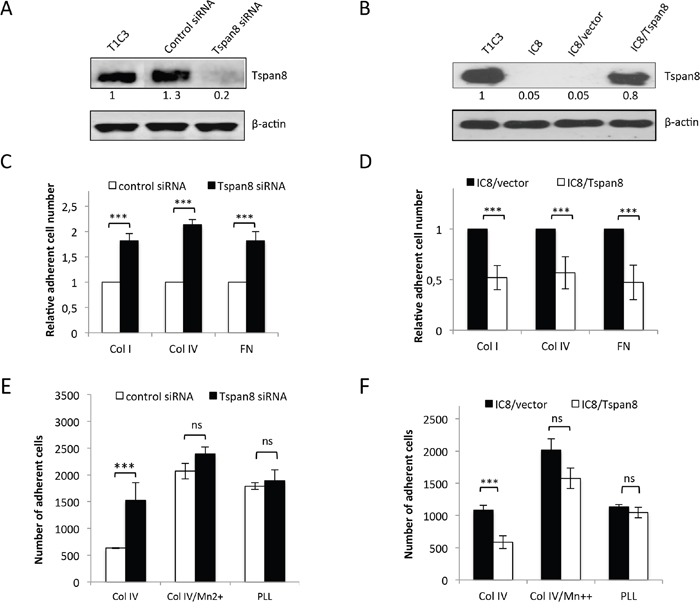
Tspan8 weakened melanoma cell-matrix anchorage **A**. Western blot showing the Tspan8 expression 48 hours after transfection of invasive T1C3 cells with siRNA targeting Tspan8 (Tspan8 siRNA) or non-targeting siRNA (control siRNA). **B**. Western blot analysis of Tspan8 expression in non-invasive IC8 cells stably transduced with empty (IC8/vector) or Tspan8 (IC8/Tspan8) expression vectors. (A and B.) Band intensities were normalized to that of β-actin, used as an internal loading control. **C-F**. These cells were serum-starved for 12 hours before seeding on collagen IV (col IV), fibronectin(FN) or poly-L-lysine (PLL)-coated plates and treated with or without manganese (Mn2+) in serum-free media. **C** and **D**. The number of adherent cells counted on the entire well surface was normalized to the value from control cells (n=6, mean ± SEM). E and F. The number of adherent cells was the mean ± SD from a representative experiment (n=4, each in quadruplicate). ***, p < 0.001, student t test.

To determine whether the Tspan8-mediated invasion of melanoma cells [23] may result from a defect in cell-matrix anchorage, we knocked-down Tspan8 expression in invasive cells by the previously validated SMART pool siRNA [23]. We showed that efficient Tspan8 silencing (Figure 1A) resulted in a significant 1.8 to 2-fold increase in cell adhesion to collagen I, collagen IV and fibronectin (Figure 1C) (p < 0.001). This effect was not dependent on immobilized ligand density since it was observed over a wide range of concentrations (2.5 to 40 μg/ml; Supplementary Figure 1). Conversely, ectopic expression of Tspan8 in non-invasive cells (at levels comparable to that of invasive cells; Figure 1B) resulted in a 2-fold reduction in cell adhesion to matrix components (Figure 1D) as compared to control cells (p < 0.001). Similar results were observed with another clone expressing ectopic Tspan8 (not shown). Importantly, the impact of Tspan8 silencing on adhesion was abrogated when using the poly-L-lysine (charge interactions only) as a substrate (Figures 1E and 1F), suggesting a possible involvement of integrins in Tspan8-mediated function. Indeed, pretreatment with Mn^2+^, a well-known potent integrin activator [24] abolished the difference in adhesion properties between Tspan8-positive and Tspan8-negative cells on collagen IV (Figures 1E, 1F), fibronectin or collagen I (not shown). These results indicate that Tspan8 expression down-regulates cell-matrix anchorage mainly through integrins.

### Tspan8 negatively regulates melanoma cell-matrix anchorage through β1-integrins, without affecting their level or pattern of expression

**Figure 2:**
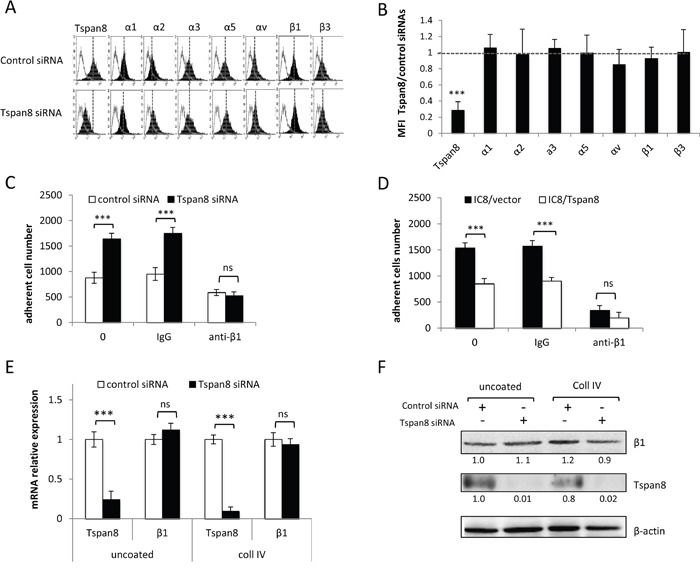
Tspan8-reduced β1 integrin-mediated adhesion was independent of β1 transcript and protein expression level **A** and **B**. T1C3 cells transfected with control or TSPAN8 siRNAs were cell surface stained with the indicated mAbs.(A) Data from one representative experiment out of five showing flow cytometry profiles of cells expressing or not Tspan8. Filled histograms represent specific staining and open histograms show isotype-matched control antibody. (B) The value represent the mean MFI ratio of Tspan8-deleted cells to Tspan8-expressing cells ± SD of 5 separate experiments. **C** and **D**. Tspan8-depleted (C) and Tspan8-ectopically expressing (D) cells were treated or not with a function-blocking β1 integrin (P4C10) or control (IgG) antibodies, seeded on collagen IV-coated plates and subjected to adhesion assays (n=4, mean ± SD). ***, p < 0.001, student t test. **E** and **F**. Cells silenced or not for Tspan8 were seeded onto plates coated or not with collagen IV and subjected to QPCR analysis (E; n=3, mean ± SD) and Western blotting (F). β-actin was used as a loading control. Band intensities were quantified and normalized to β-actin signal (representative of 3 independent experiments).

Attenuation of integrin-mediated adhesion by Tspan8 expression might result from reduced cell surface expression of specific integrin subunits and/or changes in integrin repertoire. We thus compared cell-surface expression of a panel of integrin subunits in invasive cells silenced or not for Tspan8. In both conditions, cells expressed high levels of α1, α3, α5 and β1 subunits, very low levels of α2, αv and β3 subunits and no detectable amounts of α4, α6, β4, β6 or β7 subunits (Figures 2A and 2B), even when cells were seeded on collagen I, IV or fibronectin (not shown). This was corroborated by comparable surface levels of α1, α2, α3, α5, αv, β1 and β3 integrin subunits between non invasive cells expressing ectopic Tspan8 and control cells (not shown).

The Tspan8-mediated effect was not limited to a specific type of matrix protein and the β1 integrin subunit, common to both fibronectin and collagen receptors, was predominantly expressed in melanoma cells (Figure 2A). We therefore investigated whether Tspan8 effect on cell-matrix anchorage might be mediated through β1 integrins. A function-blocking anti-β1 integrin subunit strongly decreased the elevated anchorage of cells devoid of Tspan8 to a level comparable to Tspan8-expressing cells, on both collagen IV (Figure 2C-D) and fibronectin (not shown). Moreover, mRNA (Figure 2E) and protein (Figure 2F) levels of β1 integrin subunit were unaltered after Tspan8 silencing in cells adhering to uncoated or collagen IV-coated wells. In conclusion, melanoma cell-matrix anchorage was mainly regulated by β1 integrins, and Tspan8-weakened β_1_-dependent adhesiveness was not mediated by variations in the cell surface repertoire or protein levels of integrin subunits.

### Tspan8 forms a co-immunoprecipitable complex with β1 integrin subunit and regulates its ligand binding avidity

**Figure 3:**
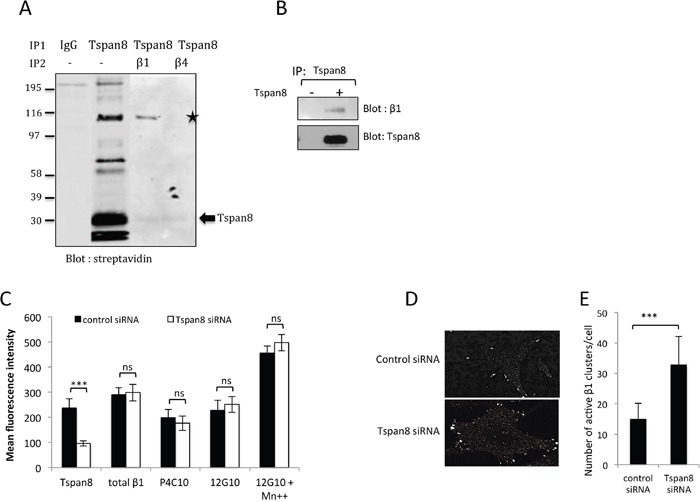
Tspan8 is in a common co-immunoprecipitable complex with β1 integrin and modulates its clustering rather than its affinity **A**. T1C3 melanoma cells were surface biotin-labeled before lysis with Brij 97, immunoprecipitated with Tspan8 mAb or IgGcontrol and immunoblotted with HRP-conjugated streptavidin. Eluted co-immunoprecipitated proteins with anti-Tspan8 (IP1) were identified by a second round of immunoprecipitations using a β_1_ integrin mAb or an irrelevant anti-β_4_ integrin mAb (IP2) and visualized by western blotting using a streptavidin-HRP secondary antibody. The asterisk indicates a streptavidin-reactive band: β_1_ integrin. **B**. Cells expressing or not Tspan8 were lysed in Brij 97, immunoprecipitated with Tspan8 mAb and examined by western blotting using β1 integrin and Tspan8 mAbs. **C**. T1C3 cells transfected with control or Tspan8 siRNAs were cell-surface stained for total β_1_ integrin (MAR4 mAb), inactive (P4C10 mAb) and active (12G10 mAb) β_1_ integrin epitopes, after treatment with Mn^2+^ and analyzed by flow cytometry. Data were expressed as mean MFI values ± S.D (n=4). ***, p < 0.001, student t test. (**D** and **E**) Cells seeded on glass coverslips coated with collagen IV were fixed 6h later and stained for active β1 using 12G10 mAb. D. Representative images of T1C3 cells transfected with control siRNA or Tspan8 siRNA (n=3). Scale bar is 10μm. E. Quantification of the mean number of active β_1_ integrin clusters per cell (40 cells per experiment, n =3). ***, p < 0.001, student t test.

To investigate whether Tspan8 could be part of a β1 integrin complex that might influence cell-matrix anchorage, we immunoprecipitated Tspan8 from invasive melanoma cells after surface biotinylation. One of the most prominent bands observed corresponded to the molecular weight of the β_1_ integrin subunit, which was identified by sequential immunoprecipitations, using first anti-Tspan8 antibody and then anti-β1 integrin antibody (Figure 3A). Integrin β_1_ immunodetection in Tspan8-immunoprecipitates from non-biotinylated melanoma cells definitely confirmed the association of the two proteins within a common complex (Figure 3B).

To examine whether Tspan8 may interfere with β1 integrin activation, we evaluated the accessibility of β1 to mAbs 12G10 and P4C10, known to ligate only activated or inactived β1 integrins respectively, regardless of the α subunit or ligand occupancy [25]. Total β1 integrins was assessed using a conformation-independent MAR4 antibody. Tspan8-silenced cells displayed amounts of surface β1-integrin receptors, either at high- or low-affinity state, comparable to those of Tspan8-expressing cells (Figure 3C). Furthermore, Tspan8 expression did not block the Mn^2+^-induced conformational activation of β1 integrin (Figure 3C), which is in line with the observation that the Tspan8-associated reduction of cell-matrix adhesion could be rescued by Mn^2+^ treatment (Figures 1E and 1F).

We next focused on the ability of Tspan8 to affect β1 integrin clustering by staining cells with 12G10 antibody. Tspan8 silencing increased clustering of active β1 integrins within large streak-like focal adhesions at the cell periphery compared to control cells (Figure 3D) with a 2-fold increase of the clusters per cell compared to control cells (Figure 3E; p<0.001). The opposite effect was observed when comparing cells expressing ectopic Tspan8 to control cells (not shown). Overall, Tspan8 expression attenuates melanoma cell-matrix anchorage by modulating β_1_-ligand binding, mainly through integrin avidity.

### Tspan8 silencing causes enhanced codistribution of β1 integrin with P-FAK at focal adhesions, without affecting FAK autophosphorylation

**Figure 4:**
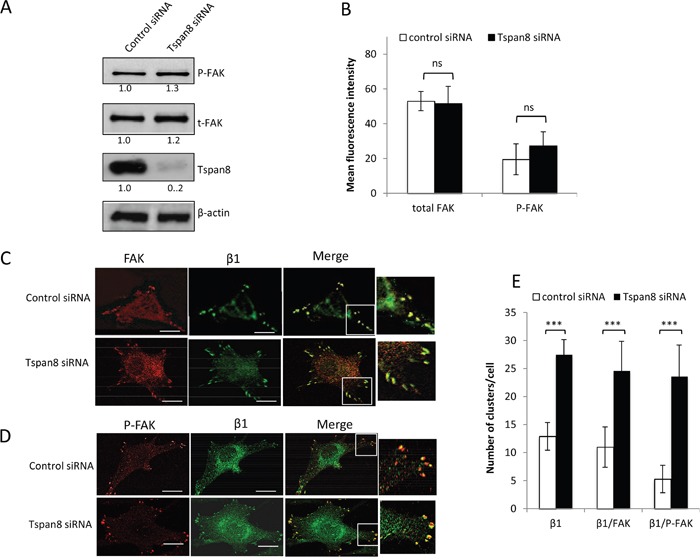
Tspan8 regulates β1 integrin clustering but does not affect phosphorylation state of FAK **A** and **B**. T1C3 cells transfected with control or TSPAN8 siRNAs were seeded on collagen IV-coated plates and subjected to western blot (A) and flow cytometry (B) for analysis of total FAK (t-FAK) and phospho-FAK (P-FAK) expression level. (A) Bands intensities were quantified and normalized to that of β-actin (representative of 3 independent experiments). (B.) The value represents the mean MFI ± SD of three independent experiments. **C-E**. T1C3 cells silenced or not for Tspan8 were plated onto collagen IV-coated coverslips and costained with FAK (red) and 12G10 (green) mAbs or P-FAK (red) and 12G10 (green). (C and D). The merged representative confocal image shows colocalization (yellow). Inset, enlarged areas of peripheral adhesion staining. Scale bar is 10 μm. (E). The number of clusters per cell containing active β1 integrin colocalized or not with FAK was quantified. Data shown represent the mean ± SD for at least 45 cells per experimental group encompassing at least three independent experiments.

Integrin clustering induced by cell-matrix attachment results in autophosphorylation of FAK on tyrosine 397 (P-FAK; [26]), a central focal adhesion protein recruited to the β1 integrin cytoplasmic domain and upregulated in many types of cancer, including melanoma [27]. We therefore investigated whether Tspan8-induced reduction of β_1-_integrin clustering could lead to alterations in the expression, phosphorylation status or distribution of FAK. Tspan8 silencing slightly increased the phosphorylation level of FAK in cells adhering to the matrix, but this effect was nonetheless not significant (Figure 4A and 4B; p = 0.227). Moreover, FAK colocalized with active β1-integrins at the cell-substratum interface and almost all active β1-integrins clusters were positive for FAK, regardless of Tspan8 expression (Figure 4C, merge, inset and Figure 4E). Remarkably, the majority of P-FAK spots colocalized with active β1 integrins in Tspan8-silenced cells, but not in control siRNA-transfected cells (Figure 4D, merge, inset and Figure 4E). So, loss of Tspan8 expression enhances the co-distribution of active β1 integrins with P-FAK at focal adhesions, without interfering with FAK autophosphorylation.

### Tspan8 regulates cell-matrix anchorage by impairing β1 integrin-mediated Akt activation

**Figure 5:**
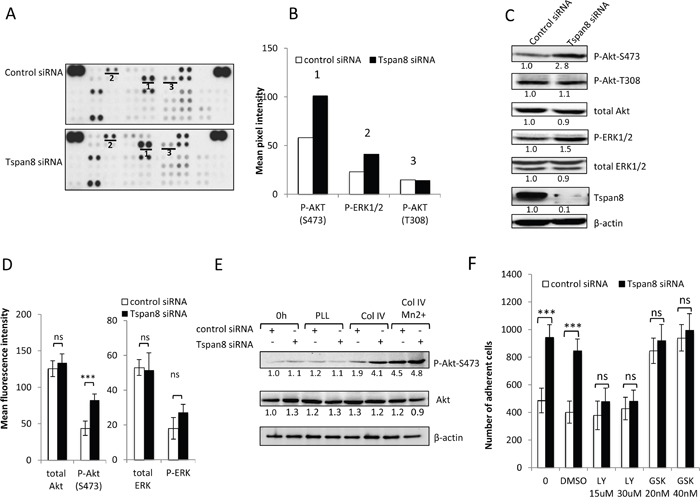
Tspan8 down-regulates β1 integrin-mediated cell adhesion through PI3K/Akt signaling **A**. Representative image of phosphokinase arrays shows the levels of phosphorylation of individual kinases present in the total protein lysates processed from control or Tspan8-siRNAs transfected cells and plated on collagen IV after 12 hours serum starvation. Each phosphorylated kinase is spotted in duplicate. The pair dots in the top right and top left corners are positive controls. Each pair of the most positive kinase dots is denoted by a numeral, the identity of the corresponding kinases is (1) P-Akt S473, (2) P-ERK1/2, (3) P-Akt T308. **B**. Mean pixel intensity of the spots measured by densitometry. **C** and **D**. The expression level of the 3 selected kinases was verified by western immunoblotting (C) and by flow cytometry (D). The results shown are representative of three independent experiments. (C) Bands intensities were quantified and normalized to that of β-actin loading control. (D) Histograms showing levels of intracellular kinases expressed as mean MFI ± SD (n= 3). ***, p < 0.001, student t test. **E**. T1C3 cells silenced or not for Tspan8 were plated on poly-L-lysine (PLL) or collagen IV (col IV), treated or not with Mn ^2+^. Cell lysates before (0 h) or after adherence were blotted for total or phosphorylated Akt mAbs (a representative blot of 3 independent experiments). The band intensities were normalized to β-actin signal. **F**. T1C3 cells transfected with control or Tspan8 siRNA were treated or not with LY294002 (LY; at 15 μM, 30 μM), GSK690693 (GSK; 20 μM, 40 μM) and vehicle (DMSO) and seeded onto collagen IV-coated plates. The adherent cells were numerated. Bars represent the mean ± SD of quadruplicate samples from one representative experiment (n=4).

We next analyzed the downstream effects of impaired β1 clustering in Tspan8-expressing cells. Using a human phospho-kinase antibody array, we compared the phosphorylation status of up to 46 well-characterized kinases between collagen IV-adherent cells silenced or not for Tspan8 (Figure 5A). Surprisingly, only phospho-ERK1/2 (T202/Y204; T185/Y187) and phospho-Akt-S473 exhibited a detectable phosphorylation level that was significantly enhanced in Tspan8-depleted cells compared to control siRNA-transfected cells (Figure 5A, 5B). We confirmed the increased phosphorylation of Akt-S473 in Tspan8-silenced cells (Figure 5C, 5D) whereas the increase in ERK1/2 phosphorylation did not reach statistical significance (Figure 5C, 5D). Notably, no significant change in the phosphorylation levels of Akt-T308 (Figure 5A-5C) and FAK (not shown) was observed, strenghtening the above data (Figure 4A, 4B). Importantly, the phosphorylation of Akt on S473 was integrin β1-dependent. Indeed, Akt-S473 exhibits an elevated signal in cells plated on collagen IV when compared to cells plated on poly-L-lysine, a substrate that does not involve integrins (Figure 5E). Besides, forcing integrin activation by Mn^2+^ treatment increased Akt-S473 activation in Tspan8-expressing cells to a level similar to Tspan8-depleted cells (Figure 5E).

To investigate whether the low phosphorylation status of Akt-S473 in Tspan8-expressing cells contributed to reduced-cell-matrix attachment, adhesion assays were performed in presence of the commonly used PI3K inhibitor LY294002, at concentrations known to prevent phosphorylation of its downstream target Akt [28]. The increased adhesiveness displayed by the Tspan8-depleted cells was abolished by LY294002 (Figure 5F; n=4, p<0.0005), with a level similar to Tspan8-expressing cells. Furthermore, treatment of cells with GSK690693, a conventional selective ATP competitor that leads to Akt hyperphosphorylation [29], abrogated Tspan8-dependent decrease of cell-matrix adhesion (n=4, p< 0.0001). The effects of these two inhibitors on adhesion were unlikely to be due to a change in cell-death phenotype (Supplementary Figure 2A). Overall, these results indicate that the weakened adhesion of Tspan8-expressing cells is mediated through an impaired β1 integrin-mediated Akt activation.

### Tspan8 modulates β1 integrin-dependent adhesion and clustering through ILK activity

**Figure 6:**
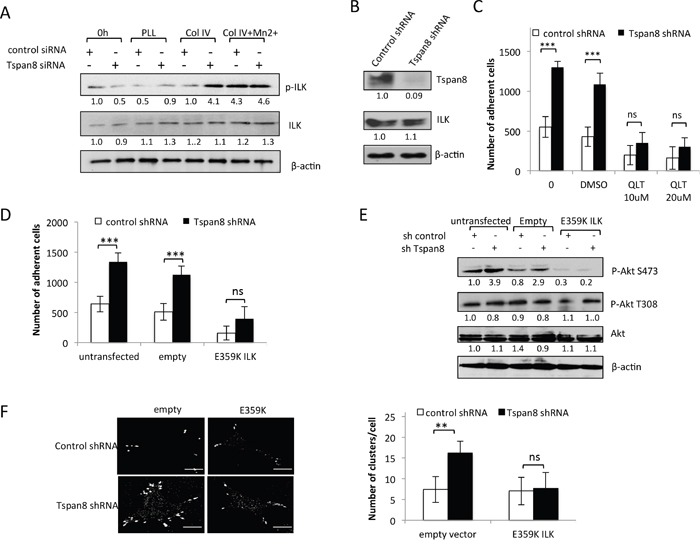
Tspan8 silencing reduces ILK activity with a concomitant decrease in β1 integrin-dependent adhesion and clustering **A**. T1C3 cells silenced or not for Tspan8 were plated on poly-L-lysine (PLL) or collagen IV (col IV) with or without Mn^2+^ and allowed to adhere for 2 h. Cell lysates before (0 h) or after adherence were analyzed by Western blot with total or phosphorylated ILK mAbs (representative blot of 3 independent experiments). The band intensities were normalized to β-actin signal. **B**. Western blot analysis of Tspan8 and ILK in invasive T1C3 cells stably transduced with control or Tspan8 shRNAs expressing plasmids. β-actin was used as a loading control. Of note, Tspan8 knockdown had no effect on the ILK protein expression levels. **C**. These cells were pre-treated or not with QLT0267 at 10 μM or 20 μM or vehicle (DMSO), plated on collagen IV-coated plates and subjected to adhesion assay. Bars represent the mean number of adherent cells ± SD from a representative experiment (n=2, each in sixplicate). ***, p < 0.001, Student t test. **D-F**. T1C3 cells stably silenced for Tspan8 were transfected or not with empty or ILK-E359K plasmids, plated onto collagen IV-coated plates and subjected to adhesion assays (D) western blot analysis (E) and microscopy (F). (D) Bars represent the number of cells that adhere to collagen IV-coated plates (mean ± SD of sixplicate samples from one representative experiment; n=3). (E) Representative western blot of total (Akt) and phosphorylated (P-Akt) Akt (n=3). β-actin was used as a loading control and a internal reference for band quantification. (F). T1C3 cells plated onto collagen IV-coated coverslips were stained with 12G10 mAb. Left panel, representative confocal microscopy images (n=2; scale bar: 10 μm). Right panel, mean number of β1 integrin clusters per cell ± S.D. from up to 40 cells over 2 separate experiments. **, p < 0.05, Student t test.

The weakened adhesive function of Tspan8 relies mainly on attenuated integrin-mediated Akt-S473 phosphorylation (Figure 5A–5E). The integrin-linked kinase (ILK) is a key regulator of cell-matrix adhesion, which interacts with the cytoplasmic domain of β1 integrin subunit [30] and plays a crucial role in melanoma progression [31]. ILK activation is involved in Akt phosphorylation on S473 in a PI3K-dependent manner [30]. We therefore investigated whether ILK activity could account for elevated Akt-S473 phosphorylation level in Tspan8-depleted cells. We observed a strong ILK phosphorylation (Figure 6A) that coincides with an increase of Akt-S473 phosphorylation (Figure 5E). Remarkably, Mn^2+^ treatment, which locks integrins into high-affinity status, nearly completely restored collagen IV-induced phosphorylation of ILK in Tspan8-expressing cells to the level observed in Tspan8-silenced cells (Figure 6A), as observed above for Akt-S473 (Figure 5C), whereas total ILK levels remained unchanged. These results suggest that Tspan8 may affect β1 integrin-dependent cell adhesion by modulating the ILK activity and consequently Akt-S473 phosphorylation.

We further explored the functional involvement of ILK by using a selective inhibitor of ILK, QLT0267 [32], and the dominant-negative kinase-deficient form of ILK (E359K; [33]) in invasive T1C3 cells stably depleted of endogenous Tspan8 (Figure 6B). As after transient silencing of Tspan8, stable Tspan8 depletion led to a 2-fold increase in integrin-dependent adhesion (Figure 6C; p< 0.001) and QLT0267 treatment in these cells drastically reduced cell-matrix adherence to the level measured in Tspan8-expressing cells (Figure 6C). The same phenotype was observed after LY294002 treatment (not shown). Alternatively, transient expression of ILK E359K reversed the increased cell-matrix adhesion of Tspan8-silenced cells back to the level seen in Tspan8-expressing cells (Figure 6D). The transduction efficiency of ILK E359K mutant was confirmed through the suppression of Akt-S473 phosphorylation (Figure 6E). Phosphorylation of T308 of Akt, a residue not reported as a target of ILK, was unchanged in all cells (Figure 6E). Inhibition of cell-matrix adherence was not due to changes in cell survival since the number of adherent cells treated or not with QLT0267 or transfected with ILK mutant was similar after 10 hours growth to that of control cells (Supplementary Figure 2B). Thus, Tspan8-induced reduction of cell-matrix adhesion is most likely due to low ILK activity.

ILK is thought to be a critical regulator of ß1-integrin avidity [34]. Since Tspan8 expression had a negative effect on ß1-integrin clustering, we tested whether changes of Tspan8-dependent ILK status could impact ß1 integrin clustering. Inhibition of endogenous ILK activity using dominant-negative ILK mutant decreased the number of active ß1 integrin clusters at the cell periphery of Tspan8-silenced cells to a value similar to that of Tspan8-expressing cells (Figure 6F). We conclude that Tspan8 functions as a pivotal regulator of ILK-driven ß1 integrin clustering and cell adhesion.

### Inverse correlation between Tspan8 and P-ILK expression in cutaneous melanoma xenografts and human melanocytic lesions

**Figure 7:**
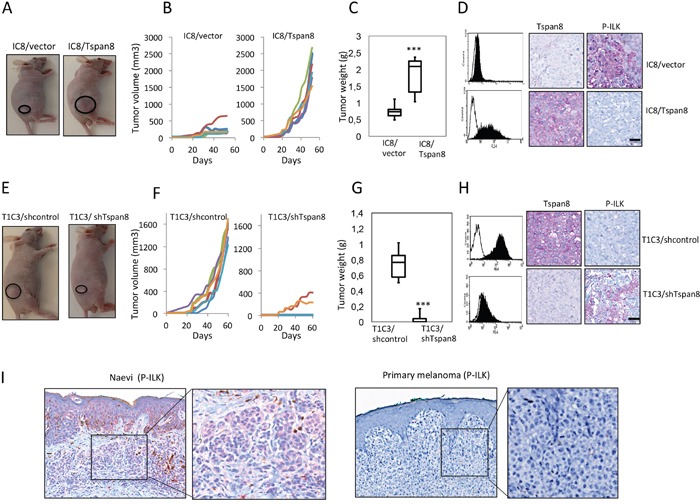
Tspan8 modulates melanoma growth in vivo and its expression correlates with downregulation of ILK phosphorylation **A-D**. IC8 cells transduced with the vector alone (IC8/vector) or Tspan8 cDNAs (IC8/Tspan8) were subcutaneously injected into the flank of nude mice (6 mice per group; n=2). (A) Representative photographs of xenografts. (B). Tumor growth curves for each mice. (C). Tumor weight was measured after mice were killed at 50 days post-injection (n=6, mean ± SEM). **p<0.005, Student's t-test. (D). The dissected xenograft tumors were subjected to flow cytometry analysis for Tspan8 expression and immunohistochemical staining with Tspan8 and P-ILK mAbs. Filled histograms represent the specific staining and open histograms show the isotype-matched control antibody. Scale bar, 100 μm. **E-H**. T1C3 cells stably expressing control (T1C3/shcontrol) or Tspan8 shRNAs (T1C3/shTspan8) were xenografted in nude mice (6 mice per group, n=2). (E) Representative photographs of xenografts. (F) Growth curves of xenografts. White symbols: volume of tumors generated from T1C3 cells transduced with Tspan8 shRNA (T1C3/shTspan8); black symbols: volume of tumors generated from T1C3 cells transduced with control shRNA (T1C3/shcontrol). (G) At day 50, the mice were euthanized and the tumors were removed and weighed. **P<0.005. (H) The dissected tumors were subjected to flow cytometry analysis for Tspan8 expression and immunohistochemical staining with Tspan8 and P-ILK mAbs. Filled histograms represent the specific staining and open histograms show the isotype-matched control antibody. Scale bar is 100 μm. **I**. Representative immunohistochemical expression of P-ILK staining in a benign nevus and primary melanoma. The square represents the area of magnification shown in the inset.

To address the *in vivo* biologic relevance of these *in vitro* findings, we orthotopically xenografted non-invasive IC8 cells stably expressing ectopic Tspan8 and invasive T1C3 cells stably silenced for endogenous Tspan8, and examined tumor development rate and P-ILK expression in subcutaneous tumors. Ectopic Tspan8 expression in non-invasive cells significantly increased tumor growth and weight (Figure 7A–7C) whereas knockdown of endogenous Tspan8 in invasive cells drastically inhibited tumor occurence and growth (Figure 7E–7G), further demonstrating the pro-oncogenic function of Tspan8 in melanoma. Flow cytometry analysis of cells prepared from the excised xenografts and immunohistochemical staining of tumors confirmed that cells retained the expressed or silenced Tspan8 (Figure 7D, 7H). Importantly, P-ILK expression was remarkably strong in Tspan8-deleted tumors and very low in Tspan8-expressing tumors (Figure 7D, 7H).

We then evaluated the clinical relevance of these results by analyzing P-ILK expression in human primary melanomas (n= 21) and benign nevi (n=13), previously selected and shown to strongly or faintly express Tspan8, respectively [23]. P-ILK staining was observed in benign nevi, although with variable intensity (strong: 9/13; weak 4/13), but never detected in primary melanomas (Figure 7H), demonstrating a mutually exclusive expression of Tspan8 and P-ILK. Thus, the *in vivo* and *in situ* findings strengthened the evidences pointing towards the ability of Tspan8 to inhibit ILK activity, allowing the appearance of a more aggressive phenotype.

## DISCUSSION

Impairment of tumor cell-matrix and intercellular communication is a prerequisite for invasion and metastasis. Although Tspan8 has emerged as a key regulator of epithelial cell-cell interactions, mostly through interaction with α6β4 integrin [35], and E-cadherin [20], very few is known for Tspan8 role in regulating cell-matrix adhesion. To our knowledge, only one report showed that Tspan8 silencing in HT29 colon cancer cells led to increased adhesiveness to laminins [21]. However, the signaling events by which Tspan8 regulates integrin functions have not yet been addressed.

Our data are the first to establish Tspan8 as a negative regulator of ILK activity, thus inhibiting β1 integrin-mediated anchorage to matrix required for allowing melanoma invasion. Importantly, the effects of Tspan8 on β1 integrin-dependent cell adhesion was observed regardless of the type of matrix protein (fibronectin or collagens), implying that this effect is not restricted to specific α/β1-integrin heterodimers. Indeed, a blocking antibody specific to the β1 subunit drastically diminished adhesion to all these different ligands. The effect was also shown to be due to reduced integrin β1-ligand binding through avidity (clustering) rather than to matrix affinity (conformation) or expression level.

Clustering of ligated integrins leads to the formation of focal adhesions, establishing a platform that regulates signaling events. FAK and ILK kinases are the two major adaptors that form a complex with the cytoplasmic tail of β1 integrin subunit and intracellularly propagate the signal transduced from adhesion sites. Interestingly, Tspan8 ablation significantly affected ILK activation in melanoma cells adhering to the matrix but not FAK autophosphorylation. This was associated with an enhanced phosphorylation status of the ILK downstream target Akt, and a concomitant increase in β1-integrin clustering. It is noteworthy that Mn^2+^ treatment enhanced the β1-dependent cell adhesion capacity of cells expressing Tspan8 to the same level as in Tspan8-negative cells, and triggered a concomitant increase in the phosphorylation status of ILK and Akt-S473. These effects were not observed when cells were plated on poly-L-lysine, a substrate that does not engage integrin, demonstrating the integrin specific liability of this process.

ILK is a key upstream regulator of Akt phosphorylation on S473 that modulates cell-matrix anchorage [36, 37]. Although T308 phosphorylation is sufficient for Akt activation, S473 phosphorylation is required for optimal Akt activity. The fact that Tspan8 expression interfered only with Akt-S473 and not with Akt-T308 phosphorylation suggests that Tspan8 restricts full Akt activity in melanoma cells through inappropriate ILK activation, leading to attenuation of β1-integrin adhesive function. ILK interacts with Rictor to mediate Akt-S473 phosphorylation in different cell types [38] and Rictor may interact with Tspan8 in glioma cells [39]. Although the implication of Rictor in melanoma cells awaits further studies, we clearly demonstrate that in the absence of Tspan8, ILK-dependent Akt-S473 phosphorylation in response to ligation of β1 integrins occurs efficiently, leading to integrin clustering. Blockade of PI3K or ILK signaling by the pharmacologic inhibitors LY294002 or QLT0267 resulted in reduced cell-matrix interaction, as did the expression of the dominant negative ILK E359K. Overall, our data demonstrate that Tspan8 modulation of β1-integrin function occurred mainly through ILK/Akt regulatory axis, resulting in a low degree of β1-integrin clustering at the cell surface leading to reduced cell-matrix anchorage.

Although numerous studies associated ILK activity with the conformational status of β1**-**integrins, few focused on its involvement in integrin clustering. We showed here that ILK E359K mutant caused a decrease in the number of β1-integrin clusters, leading to attenuated cell-matrix adhesion. It was previously reported that this ILK mutant acts in a dominant-negative fashion in several ILK signaling-dependent processes, in particular in cell adhesion [40, 41], probably due to its inability to bind paxillin [42], a scaffolding adaptor known to form bridges between integrins and FAK. Here, we did not observe any significant changes in FAK autophosphorylation between cells silenced or nor for Tspan8. Since the effects of ILK E359K mutant on matrix-adhesion in Tspan8-negative cells mimicked those obtained in Tspan8-expressing cells, it seems unlikely that Tspan8 prevents interaction of β1-integrins with FAK-paxillin, especially as Tspan8 loss has no impact on paxillin phosphorylation, as observed on the kinase array (not shown). A recent study reported that siRNA-knockdown of Tspan8 in glioma cells reduced FAK-Y397 phosphorylation and decreased *in vitro* cell proliferation rate [43]. Moreover, Tspan8 exerts a pro-migratory function of epithelial cells through its association with the laminin-binding receptor α6β4, leading to FAK activation [12, 16]. Since the modulation of the expression of Tspan8 does not affect the *in vitro* proliferation and motility of melanoma cells [23], it may be postulated that Tspan8 specifically modulates cell-matrix adhesion and invasion phenotypes in cutaneous melanoma, whereas it regulates proliferation and migration in other cancers, independently and dependently of FAK, respectively.

Reduced clustering of ligated integrins strongly influences their function and signaling [44], and may arise as a result of altered lateral diffusion of integrins [45]. Using fluorescence photobleaching experiments, we previously reported that high- and low-agressive melanoma cells displayed clear-cut differences in the lateral motion of glycoproteins in the plasma membrane [46]. Furthermore, it has been reported that the tetraspanin CD151, known to upregulate melanoma cell motility [47], was able to disturb the mode of integrin diffusion at the plasma membrane [48]. In this context, and given that integrin clustering seems to be driven mainly by the β-subunit rather than the α-subunit [49], it is tempting to speculate that Tspan8, by interacting laterally with integrins of the β1 subfamily, restricts their diffusion and thereby their clustering, regardless of the α-associated subunit. This is consistent with the fact that Tspan8 formed an immunoprecipitable complex with β1 integrins at the surface of invasive melanoma cells and reduced adhesion to a wide range of ligands.

Tspan8 expression in invasive melanoma cells was responsible for inhibition of ILK phosphorylation and its downstream target Akt-S473, concomitantly with reduced adhesiveness. These *in vitro* findings have *in vivo* significance, since we found that the expression of Tspan8 is inversely correlated with ILK phosphorylation in melanoma xenografts and in human melanocytic lesions. These data highlight an inverse relationship between Tspan8 expression and ILK activity with respect to disease progression, further supporting an involvement of Tspan8-ILK regulation in cutaneous melanomas. In summary, using a combination of *in vitro, in vivo and in situ* approaches, we uncovered a hitherto unappreciated function of Tspan8 in negatively regulating β1 integrin-dependent adhesion and avidity through ILK, which sets off the aggressiveness of melanoma cells by decreasing adhesive interaction with the surrounding matrix environment and promoting tumor escape. Since invasive Tspan8-positive cells also expressed peanut agglutinin lectin (PNA)-binding sites [23], reported to be associated with a propensity for dermal invasion [50], *in vivo* metastasis [51], and with poor clinical outcome of patients [52, 53], it is conceivable that Tspan8 identifies tumor cell subpopulations with low adhesive potential, able to escape from the primary tumor and move unhindered in the matrix environment to circulate and metastasize. Hence, Tspan8 may represent a promising target for both early detection and anti-invasive therapies in cutaneous melanoma. Indeed, targeting Tspan8 to switch from a “non-sticky” to a “sticky” state may provide a means to prevent the spread of aggressive cells and thus, metastasis. This strategy is consistent with recent data identifying Tspan8 as a blood biomarker candidate for early detection of human colon cancer [54], and demonstrating that Tspan8-specific antibodies reduce the incidence of ovarian cancer metastasis [55].

## MATERIALS AND METHODS

### Antibodies

The monoclonal antibodies (mAbs) against integrins: beta1 (MAR4 clone), alpha1 (SR84 clone), alpha2 (12F1-H6 clone), alpha3 (C3 II-1 clone), alpha4 (9F10 clone), alpha5 (VC5 clone) were purchased from BD Pharmingen (Le Pont de Claix, France) and alpha V (M9 clone), beta3 (VI-PL2 clone), beta5 (P1F6 clones), beta6 (10D5 clones) from Millipore (Mannheim, Germany). Were also used for Western blotting and flow cytometry the antibodies against P-ERK (sc-738), ERK (C-16) and FAK (A-17 clone) from Santa Cruz, CA; Y397-FAK (44-624 G; Invitrogen); P-ILK (Pierce); ILK (#3862), Akt and P-Akt (Cell Signaling); P4C10 and 12G10 (BD Biosciences, Franklin Lakes, NJ). Mabs for human Tspan8 (TS29 clone) was a generous gift from Dr C. Boucheix (Inserm U602, Villejuif).

### Cell lines and culture

Non-invasive IC8 and invasive T1C3 human melanoma clones were selected from the same parental cell line and described in [23]. T1C3 cells expressed high level of Tspan8 [23], invade dermis in skin reconstructs [50], and promote lung metastases in immunosuppressed newborn rats [51]. Conversely, IC8 cells did not express Tspan8, are unable to form metastasis in animals and to invade dermis in skin reconstructs [50, 51]. They were cultured under standard conditions and tested as mycoplasma-free as previously described [23].

### Transient Tspan8 silencing

ON-TARGET plus human TSPAN8 siRNA SMARTpool (Dharmacon, Chicago, IL, USA), was previously selected based on silencing potency and lack of off-target effects on T1C3 melanoma cells [23]. Briefly, T1C3 melanoma cells were transfected with 5 nM of TSPAN8 siRNA SMARTpool or control ON-TARGET plus non-targeting pool siRNA. Tspan8 knockdown was checked using QPCR, flow cytometry and western blot.

### Transient expression of ILK-E359K

T1C3 melanoma cells stably depleted or not for endogenous Tspan8 were transiently transfected with 250μg of empty or ILK-E359K (dominant-negative) pcDNA6-myc vector using JetPei (Ozyme), according to the manufacturer instructions. This vector provided by Dr S. Dedhar (British Columbia Cancer Research Center, Vancouver, Canada) has been previously described in detail [33]. Cells were harvested 48h after transfection and subjected to adhesion assay, confocal analysis or Western blotting.

### Stable Tspan8-positive and negative clones

Stable cell clones expressing ectopic Tspan8 (IC8/Tspan8) were generated by transfecting IC8 cells with pCDNA3-Tspan8 (a generous gift from C. Boucheix; Villejuif, France) followed by G418 antibiotic selection. Endogenous Tspan8 was silenced in T1C3 cells after puromycin selection of clones stably expressing HuSH 29-mer Tspan8-shRNA (Origene Technologies, Rockville, MD, USA). Successful expression or knockdown of Tspan8 was confirmed by QPCR, immunoblotting and flow cytometry.

### Cell attachment assay

Ninety-six-microwell plates were coated overnight at 4°C with fibronectin, collagen IV, collagen I or poly-L-Lysine (10 μg/ml; BD Biosciences,). The cells were detached after over-night serum starvation and seeded on wells previously blocked with 1% bovine serum albumin at 6 × 10^4^ cells/well density for 30min at 37°C. Additional experiments were accomplished after a pre-incubation of 30min with saturating concentrations of function-blocking anti-β1-integrin, control antibodies, pharmacological inhibitors, in presence or absence of 1mM Mn^2+^. After staining with DAPI, images of the total well surface area were acquired using Zeiss Axiovert 200 microscope and a Coolsnap HQ2 camera with a 10x objective. The total number of adherent cells per well was counted using NIH Image J software.

### Flow cytometry

Cell surface labeling and intracellular staining were performed as previously described [23, 56]. Results were normalized by subtracting the MFI of the appropriate control and expressed by the mean (3 independent experiments) of MFI +/- SD or the ratio of MFI of Tspan8-positive cells /MFI of Tspan8-negative cells.

### Phospho-kinase array

The relative site-specific phosphorylation of 46 kinases was analyzed using the Human Phospho-Kinase Array Kit from R&D Systems according to the protocol provided by the manufacturer. Briefly, 48h after transfection of T1C3 cells with control or Tspan8 siRNA, cells were serum-starved for 12h, seeded on collagen IV-coated flask for 2h and lysed with the recommended lysis buffer. The chemiluminescence signal intensity of individual spot was analyzed using Quantity One 1-D Analysis Software (BIO-RAD, Marnes-la-Coquette, France).

### Immunoprecipitation experiments

Cell surface biotinylation and immunoprecipitation procedures were performed as previously described [57]. Briefly, cells were incubated with PBS/2.5 mg EZ-LinkSulfo-NHS-SS-Biotin (Pierce, Rockford, IL, USA) at 4°C for 1 h and the biotinylation reaction was terminated by addition of Tris-HCl 50 mM, pH 7.5. Cells were lysed using 1% Brij97 (Sigma, St Louis, MO) buffer. After 30 min at 4°C, insoluble material was removed and the cell lysate was precleared for 2h using heat inactivated goat serum and protein G sepharose beads (GE Healthcare, England). The cell extracts were then incubated with control or specific mAb directly conjugated to sepharose 4B for 2 h, washed and eluted with Laemmli buffer. For re-immunoprecipitation experiments, associated proteins were eluted using a 1% Triton X100 buffer and then precipitated a second time with the indicated mAb-conjugated sepharose beads for 1 hour at 4°C.

### Western blotting analysis

Immunoblotting were carried out as previously described [23]. Densitometric analysis was performed using Image J software (National Institutes of Health, USA). Data are presented as values directly below the blots and are representative of at least three independent experiments.

### Confocal microscopy

Melanoma cells allowed to attach on collagen IV-coated circular glass coverslips for indicated times (1h-12h) were fixed with PBS/4% paraformaldehyde and 5% sucrose, permeabilized with 1% Brij 97 and blocked with 10% heat-inactivated goat serum. Staining of cells with indicated antibodies was performed overnight at 4°C. After incubation with secondary fluorochorme conjugated antibodies, the coverslips were mounted in the antifade reagent Mowiol. Fluorescence staining was visualized with LSM510META laser scanning confocal microscope (Carl Zeiss, Jena, Germany) using a Zeiss Plan-Apochromat *63x*/1.4 oil *objective*. Immunofluorescence images were acquired at high confocality (pinhole = 1 Airy unit) to achieve the thinnest possible optical slices at the substrate–cell interface. To determine the number of clusters for each experiment, we considered random fields with a total cell number over 50 cells per experiment (n =2).

### Animals and tumor xenografts

Six- to 8-weeks old male Swiss Nude mice (Charles River, l'Arbresle, France) were housed at the animal facilities of the UMR990 (Clermont-Ferrand, France), under specific pathogen-free conditions, in accordance with the guidelines for the Care and Use of Laboratory Animals (National Research Council, 1996) and European Directive (86/609/EEC). Cells stably expressing or silenced for Tspan8 were injected subcutaneously into mouse flank region (1 × 10^6^ cells per flank; 6 mice for each group; n=2). Tumor growth was monitored 3 times per week; measured using a caliper. Tumor volume was calculated according to the formula: *V* = 0.52 × *d*^2^ × *D* (*D*=largest diameter, d=smallest diameter). 7 to 8 weeks after, mice were sacrificed and tumors were weighed before being fixed in 4% formalin, embedded and sectioned for histology and immunohistochemistry analysis. In some experiments, melanoma cells were mechanically dissociated from tumors, immediately stained for Tspan8 and analyzed by flow cytometry.

### Human samples

Formalin-fixed, paraffin-embedded sections selected from non-dysplastic nevi (n = 13) and primary melanomas (n = 21) were previously shown to be Tspan8-negative and Tspan8-positive, respectively [23]. These lesions were obtained from the Pathology Department of the Centre Léon Bérard (Lyon, France) according to an approved Institutional Review Board protocol. 4-μm tissue sections were screened for P-ILK protein expression by a biotin-streptavidin-amplified technique with an alkaline phosphatase kit (Dako-LSAB2-System, DAKO, Hamburg, Germany) using an automated immunostainer (Ventana, Tucson, AZ), as previously described [23].

### Statistical analysis

Results were analyzed for statistical significance using Student’s t-test. P-values <0.05 were considered statistically significant (*P<0.05, **P<0.01, ***P<0.001).

## SUPPLEMENTARY MATERIALS FIGURES AND TABLES


