# Supplementary material for: Tetraspanin 8 is a novel regulator of ILK-driven β1 integrin adhesion and signaling in invasive melanoma cells
Source: Oncotarget. 2017 Feb 4;8(10):17140–55. doi: 10.18632/oncotarget.15084 (PMC5370029; doi:10.18632/oncotarget.15084)
Supplement: Supplementary file 1 [file oncotarget-08-17140-s001.pdf]

Tetraspanin 8 is a novel regulator of ILK-driven β1 integrin adhesion and signaling in invasive melanoma cells

SUPPLEMENTARY FIGURES

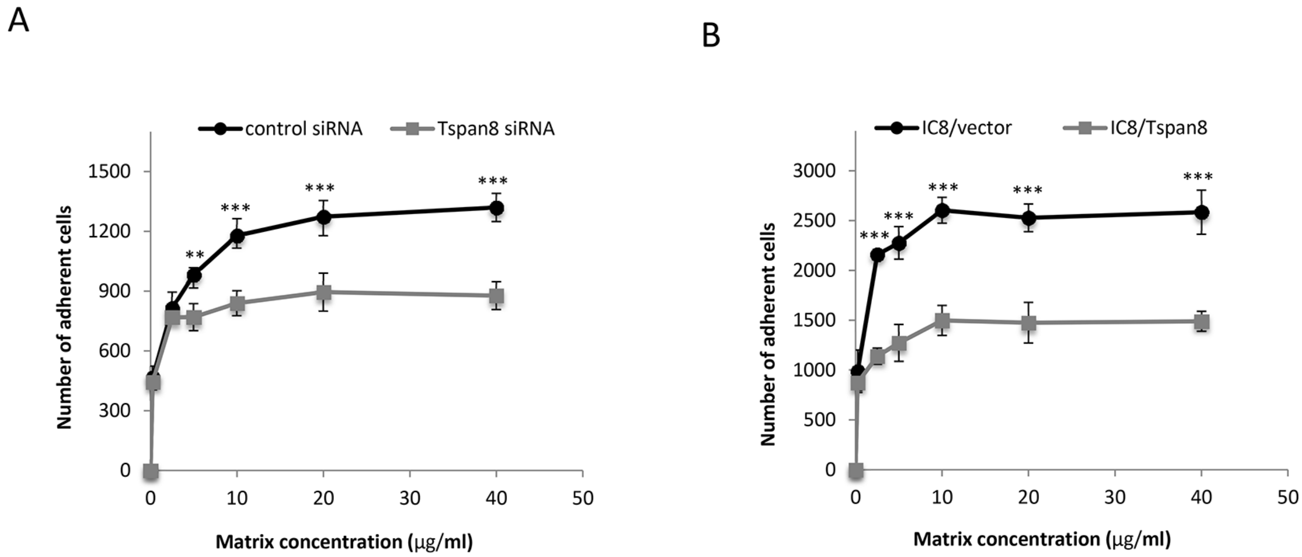

Supplementary Figure 1: Tspan8 reduced melanoma cell-matrix anchorage at different density of immobilized ligands. **A.** invasive T1C3 cells transfected with Tspan8 (Tspan8 siRNA) or non targeting (control siRNA) siRNAs and **B.**, non invasive IC8 cells stably transduced with empty (IC8/vector) or Tspan8 (IC8/Tspan8) expression vectors were serum-starved for 10 hours and then seeded for 30 minutes on plates coated with different concentrations of type IV collagen. The number of adherent cells counted on the entire well surface was the mean ± SD from a representative experiment (n=2, each in quadruplicate). ***, $p < 0.001$, student t test. **$P<0.005$, Student's *t*-test

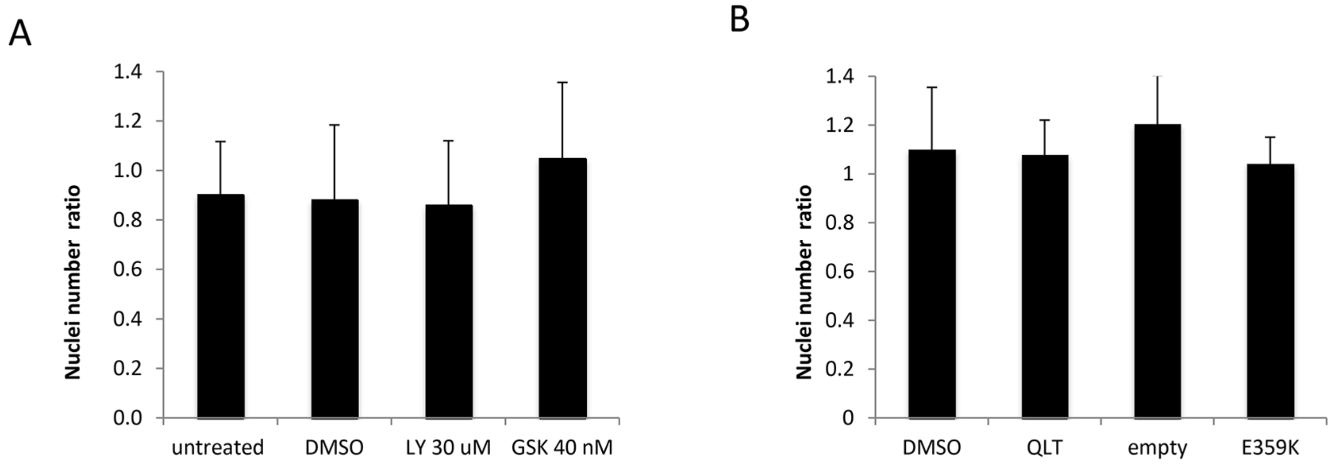

Supplementary Figure 2: Inhibition of PI3K, Akt and ILK activities did not affect cell-death phenotype. A. T1C3 cells transfected with control or Tspan8 siRNA, treated or not with LY294002 (LY; at 15 µM, 30 µM), GSK690693 (GSK; at 20 µM, 40 µM) or vehicle (DMSO) were seeded onto collagen IV-coated plates. After 10 h, cells were fixed and labeled with Hoechst 33258 for automated nucleus counts on images taken at x4 magnification (1 image per well). Bars represent the ratio of nuclei number of Tspan8 siRNA/ control siRNA (mean ± SEM, n = 3). **B.** T1C3 cells stably transduced with control and Tspan8 shRNAs expressing plasmids were treated with DMSO, QLT0267 (QLT) or transfected with empty or ILK-E359K plasmid, and plated onto collagen IV-coated plates during 10 h under serum-free conditions. Cells were then fixed and labeled with Hoechst 33258 for automated nucleus counts on images taken at x 4 magnification (1 image per well). Bars represent the mean nuclei number of adherent cells ± SD from a representative experiment (n = 2, each in sixplicate).
